# Iridoid Glycosides Isolated from *Bellardia trixago* Identified as Inhibitors of *Orobanche cumana* Radicle Growth

**DOI:** 10.3390/toxins14080559

**Published:** 2022-08-17

**Authors:** Gabriele Soriano, Antonietta Siciliano, Mónica Fernández-Aparicio, Antonio Cala Peralta, Marco Masi, Antonio Moreno-Robles, Marco Guida, Alessio Cimmino

**Affiliations:** 1 Department of Chemical Sciences, University of Naples Federico II, Complesso Universitario Monte S. Angelo, Via Cintia 4, 80126 Naples, Italy; 2 Department of Biology, University of Naples Federico II, Complesso Universitario Monte Sant’Angelo, Via Cintia 4, 80126 Naples, Italy; 3 Department of Plant Breeding, Institute for Sustainable Agriculture (IAS), CSIC, Avenida Menéndez Pidal s/n, 14004 Córdoba, Spain; 4 Allelopathy Group, Department of Organic Chemistry, School of Science, Institute of Biomolecules (INBIO), University of Cádiz, C/República Saharaui 7, 11510 Cádiz, Spain; 5 Department of Electronics and Computer Engineering, University of Córdoba, Campus de Rabanales, 14071 Córdoba, Spain

**Keywords:** parasitic weed, melampyroside, allelopathy, ecotoxicity, sustainable crop protection

## Abstract

*Orobanche cumana* is an obligate holoparasitic plant with noxious effects in sunflower crops. *Bellardia trixago* is a facultative hemiparasitic plant that infects ruderal plants without noxious significance in agriculture and is known to produce a wide spectrum of bioactive metabolites. The objective of this study was to evaluate the allelopathic effects of *B. trixago* on the growth of *O. cumana* seedlings. Three different extracts using solvents of increasing polarity (*n*-hexane, dichloromethane and ethyl acetate) were prepared from the flowers, aerial green organs and roots of two populations, a white-flowered and a yellow-flowered population of *B. trixago*, both collected in southern Spain. Each extract was studied using allelopathic screenings on *O. cumana* which resulted in the identification of allelopathic activity of the ethyl acetate extracts against *Orobanche* radicles. Five iridoid glycosides were isolated together with benzoic acid from the ethyl acetate extract of aerial green organs by bio-guided purification. These compounds were identified as bartsioside, melampyroside, mussaenoside, gardoside methyl ester and aucubin. Among them, melampyroside was found to be the most abundant constituent in the extract (44.3% *w*/*w*), as well as the most phytotoxic iridoid on *O. cumana* radicle, showing a 72.6% inhibition of radicle growth. This activity of melampyroside was significantly high when compared with the inhibitory activity of benzoic acid (25.9%), a phenolic acid with known allelopathic activity against weeds. The ecotoxicological profile of melampyroside was evaluated using organisms representing different trophic levels of the aquatic and terrestrial ecosystems, namely producers (green freshwater algae *Raphidocelis subcapitata* and macrophyte *Lepidium sativum*), consumers (water flea *Daphnia magna* and nematode *Caenorhabditis elegans*) and decomposers (bacterium *Aliivibrio fischeri*). The ecotoxicity of melampyroside differed significantly depending on the test organism showing the highest toxicity to daphnia, nematodes and bacteria, and a lower toxicity to algae and macrophytes. The findings of the present study may provide useful information for the generation of green alternatives to synthetic herbicides for the control of *O. cumana*.

## 1. Introduction

Approximately 1% of all angiosperms are parasites with the ability to infect other plants. Some parasitic plants are facultative parasites, capable of living autotrophically but shifting to a parasitic life form when a host is available, while others are obligated parasites requiring the infection of another plant shortly after germination. Some parasitic plants are hemiparasites with the ability to photosynthesize, while others are holoparasitic plants without photosynthetic competence, relying on their host for photoassimilates. Parasitic plants are distributed among 28 dicotyledonous families, and among them, the Orobanchaceae contains examples of parasitic species from all cases of host dependency [1,2]. Orobanchaceae contains facultative hemiparasitic plants, such as the non-weedy *Bellardia trixago* L. (syn. *Bartsia trixago* L.) with a Mediterranean origin that parasitizes the roots of ruderal species [3,4]. Orobanchaceae also contains obligate holoparasitic weeds from *Orobanche* genus from which control is limited or non-existent [5].

Among *Orobanche* species, *Orobanche cumana* Wallr. is one of the most noxious biotic stresses for sunflower crops [6]. Sunflower infection by *O. cumana* occurs in southern and eastern Europe, in the Mediterranean basin and in Asia [7]. Both types of sunflower, the oilseed type and the confectionary type, are severely affected by *O. cumana* [8]. The most feasible crop protection measures against the infection of *Orobanche* species are the cultivation of resistant varieties and chemical control [1,9]. However, for the specific sunflower problem caused by *O. cumana*, the resistant varieties are not durable, since their bred resistance is overcome by new races of *O. cumana* [10]. On the other hand, the chemical solution to control *O. cumana* is the use of imidazolinone herbicides that inhibit the enzyme acetohydroxy acid synthase [8,11]. However, the capacity to evolve imidazolinone-resistance in weeds, and the lack of alternative chemical methods for *O. cumana* threatens the sustainability of chemical control of *O. cumana* in sunflower [12]. Characterization of novel modes of allelopathic action against *O. cumana* in previously known natural compounds is an alternative solution to provide efficacy and sustainability in strategies for parasitic weed management [13].

*B. trixago* is a source of several bioactive compounds [14,15,16,17] but the screening of *B. trixago* as source of herbicidal compounds has not been performed before. Different *B. trixago* extracts have been screened for insecticidal activity [18,19]. Formisano et al. [19] demonstrated variation in insecticidal activity among different parts of *B. trixago* plants, with root extracts being significantly more active than extracts from aerial parts. In addition, Barrero et al. [16] demonstrated that qualitative and quantitative differences exist in the plant chemical composition among different populations of *B. trixago*. Here, we report the isolation and identification of iridoid glycosides—bartsioside, melampyroside and mussaenoside—with inhibitory activity on *O. cumana* radicle growth. Furthermore, the ecotoxicity of melampyroside (the most phytotoxic compound isolated) was assessed considering aquatic and terrestrial ecosystems as well as different trophic levels to reveal its toxicity and safety to environment and human health in order to be used as potent biocontrol agent.

## 2. Results and Discussion

**Figure 1:**
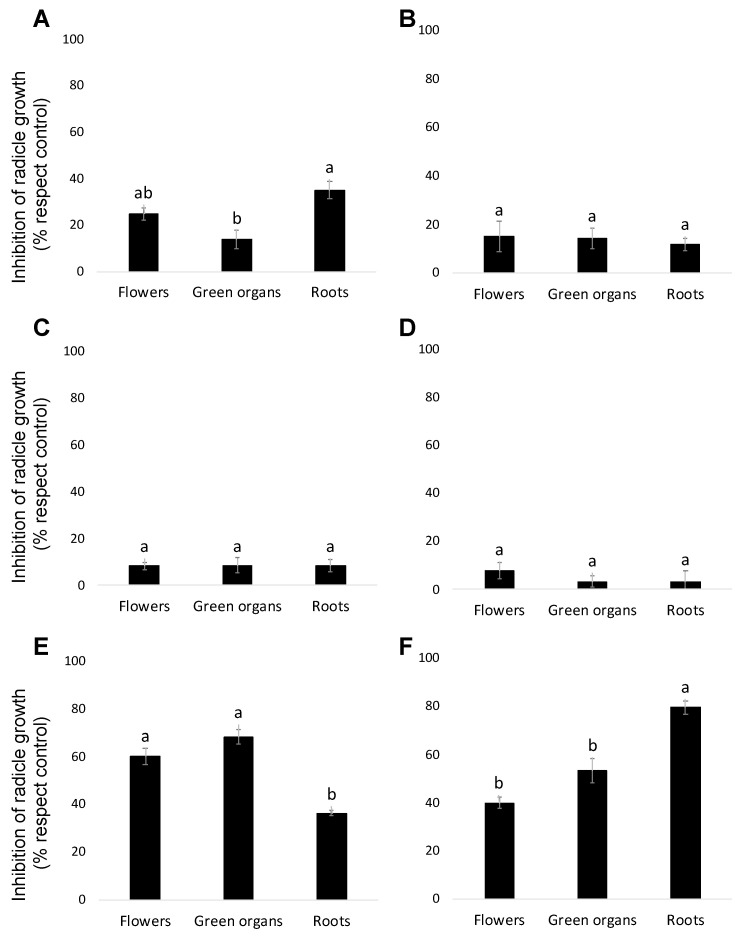
Allelopathic effects on *Orobanche cumana* radicle growth induced by extracts prepared from sequential extractions with *n*-hexane (**A**,**B**), dichloromethane (**C**,**D**), and ethyl acetate (**E**,**F**) of three types of *Bellardia trixago* organs: flowers, aerial green organs and roots of two *Bellardia trixago* populations—a white-flowered population (**A**,**C**,**E**) and yellow-flowered population (**B**,**D**,**F**). In each figure, bars with different letters are significantly different according to the Tukey test (*p* = 0.05). Error bars represent the standard error of the mean.

Three different organs (flowers, aerial vegetative green organs and roots) of two *B. trixago* populations (a white-flowered and yellow-flowered population) were extracted by maceration with a hydroalcoholic solution and then employing three different solvents of increasing polarity (*n*-hexane, dichloromethane and ethyl acetate) in sequential order as described in the Materials and Methods section. The allelopathic activity of the resulting *B. trixago* extracts was analysed at 100 μg/mL using radicle growth bioassays on *O. cumana* (Figure 1). The inhibition of radicle growth was significantly affected by the *B. trixago* population and by the solvent used for the extraction, but not by the plant organ (ANOVA, *p* = 0.022, *p* < 0.001 and *p* = 0.347 respectively). Significant effects on radicle growth inhibition were observed by the interaction of *B. trixago* population × plant organ (ANOVA, *p* = 0.002), by the interaction *B. trixago* population × solvent used for the extraction (ANOVA, *p* < 0.007) and also by the interaction plant organ × solvent used for the extraction (ANOVA, *p* < 0.009). Previously, quantitative and qualitative variations in the chemical profiles have been reported among different populations of *B. trixago* [16] and among different plant organs of *B. trixago* plants [19]. Formisano et al. [19] located the insecticidal activity in the roots of one *B. trixago* population collected in Italy, whereas aerial parts of plants of the same population were less active.

In both populations studied, the main inhibitory activity was obtained with the ethyl acetate (EtOAc) extraction (Figure 1E,F). In the white-flowered population, the strongest inhibitory activity was found in the EtOAc extract of the aerial parts, mainly in the green vegetative organs followed by the EtOAc extract of the flowers (68.31 ± 2.9% and 60.1 ± 3.4% inhibition, respectively, in comparison with control). In the yellow-flowered *B. trixago* population, the strongest *Orobanche* inhibition activity was found in the EtOAc extract of roots (inhibition average of 79.5 ± 2.9%), followed by the EtOAc extracts of aerial parts of plants, both green organs and flowers (53.5 ± 5.1% and 40.1 ± 2.5% inhibition, respectively, in comparison with control). As a result of the allelopathic screening, a preliminary qualitative evaluation of the chromatographic profiles of all EtOAc extracts was performed. This evaluation revealed the common presence of a main compound and a common pattern of secondary metabolites among the different populations and plant organs (data not showed). This fact and also the larger amount of vegetative green tissues available in the laboratory allowed the selection of EtOAc extract of green organs of the white-flowered population as the source for the isolation and characterization of inhibitors of *O. cumana* radicle growth. 

**Figure 2:**
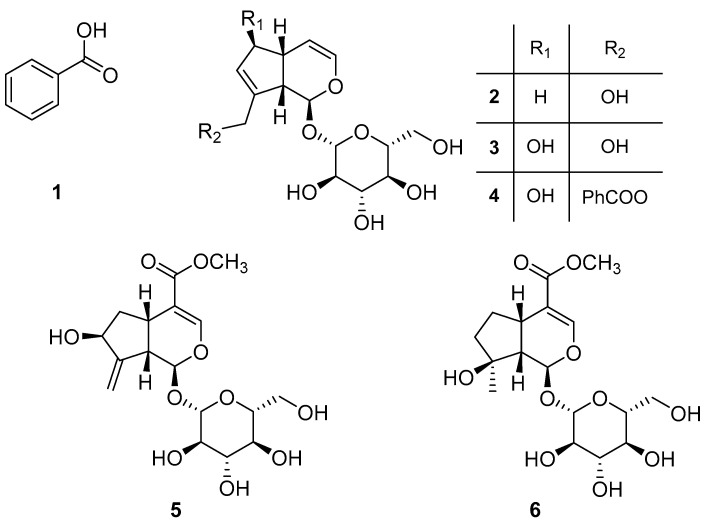
Chemical structures of benzoic acid (**1**), bartsioside (**2**), aucubin (**3**), melampyroside (**4**), gardoside methyl ester (**5**), and mussaenoside (**6**).

Thus, an amount of 189.0 g of lyophilized green organs of the white population were extracted following the procedure described in the Materials and Methods section. The sample yielded 1.45 g (0.77%) of EtOAc organic extract which was fractionated by different steps of purification by column chromatography and preparative TLCs, as reported in Scheme S1, obtaining six pure compounds which were identified as benzoic acid (**1**, 10.8 mg), bartsioside (**2**, 13.9 mg), aucubin (**3**, 12.4 mg), melampyroside (**4**, 642.3 mg), gardoside methyl ester (**5**, 2.0 mg), and mussaenoside (**6**, 6.1 mg) (Figure 2). The structures of these compounds were confirmed by NMR spectroscopy and MS, and by comparison with the data reported in the literature. Optical rotation allowed us to unequivocally identify the stereochemistry of the compounds by comparing with the values of the natural iridoids, which is a well-established family of natural products among which absolute stereochemistry was previously reported by chiroptical methods and X Ray [20,21]. Compounds **1** [16] and **2**–**6** were previously isolated from *B. trixago* [17,22] and other iridoid-containing plants [23,24,25,26,27,28,29,30].

**Figure 3:**
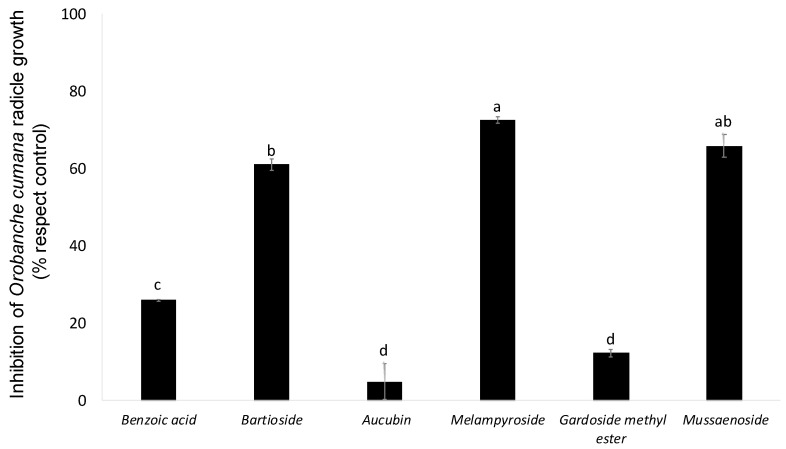
Inhibition of *Orobanche cumana* radicle growth induced by benzoic acid (**1**), bartioside (**2**), aucubin (**3**), melampyroside (**4**), gardoside methyl ester (**5**) and mussaenoside (**6**) at 100 µg/mL. Bars with different letters are significantly different according to the Tukey test (*p* = 0.05). Error bars represent the standard error of the mean.

The allelopathic effects of compounds **1**–**6** were assayed at 100 μg/mL on *O. cumana* radicles (Figure 3). Compounds **3** and **5** showed no significant inhibitory activity in the growth of *O. cumana* radicles in comparison with radicles treated with the control. On the other hand, compound **4** showed the strongest inhibition of radicle growth (72.6 ± 0.9%), followed by the inhibition activity induced by compounds **2** and **6** (61.1 ± 1.5% and 65.9 ± 2.9%, respectively). This is the first time that the inhibitory activity of *Orobanche* radicle growth has been reported in compounds **2**, **4** and **6**. Their activity was significantly higher than the activity of compound **1,** a phenolic acid with recognized weedicide activity [31]. Compound **1** showed low but significant inhibitory activity on *O. cumana* radicle (25.9 ± 0.3%), which agrees with the moderate inhibitory activity observed by a previously study on the radicles of the legume-specific parasitic plant *Orobanche crenata* [32]. Compound **1** has been previously described as a growth-regulating agent, affecting plant growth in a dose-dependent manner on different plants [33,34,35]. Previous reports describe formulations including **1** in a combination with other components as an herbicide or growth-regulating agent [36,37,38]. 

Compound **4** was firstly reported as an iridoid in *Melampyrum silvaticum* L. [39] while compounds **2**, **3**, **5** and **6** were first isolated from *B. trixago* [15], *Aucuba japonica* Thunb. [40], *Melampyrum arvense* L. [28] and *Mussaenda parviflora* Miq. [41], respectively. In the recent literature, it has been reported that compound **4** has anti-inflammatory activity [42], along with other iridoids not reported herein, which linked to a study on the potential activity of *Odontites vulgaris* against rheumatoid arthritis. In an even earlier study [43] including other iridoids and compound **4**, the latter was also found to be cardioactive in Wistar rats.

**Figure 4:**
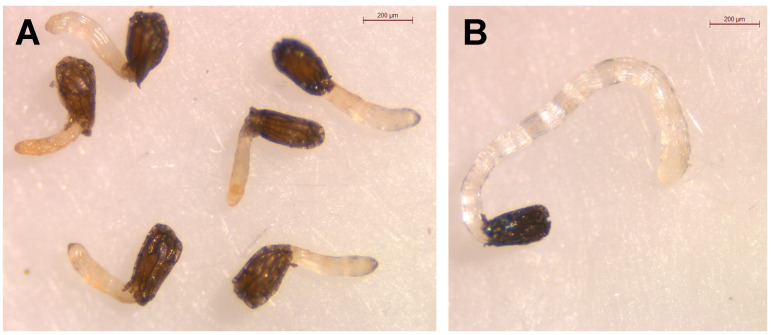
Growth of *Orobanche cumana* radicles treated with melampyroside (**4**) at 100 µg/mL (**A**) and control (**B**).

Despite the similar chemical structures of **3** and **4**, differences in their biological activities have been reported before. Compounds **4**, **1** and **3**, isolated from *M. arvense*, were reported to display antiprotozoal effects on different species, showing a certain degree of species-specificity [24]. Among the most active compounds **2**, **4** and **6** on *O. cumana* growth, compound **4** induced some degree of phytotoxicity observed as darkening in the *O. cumana* radicles (Figure 4). Compounds **3**, **4** and **6** were tested for antioxidant activity in previous reports [23,44]. Although no DPPH scavenging activity was found for these compounds, interestingly, through a β-carotene bleaching assay [23], compound **6** was found to be an antioxidant, **4** a pro-oxidant (by inducing a faster than spontaneous oxidation of β-carotene) and **3** was inactive. The pro-oxidation effect of **4** could damage the tissue of the plant explaining phytotoxicity; however, whether and how the pro-oxidant activity and the lack of antioxidant activity of **3** might be related with the observed (or not) inhibition of growth is unclear.

CLog*P* was calculated for the isolated compounds in an effort to correlate the observed inhibitory activity of compounds **1**–**6** (Table 1). Negative values were obtained for all the compounds except **1**, indicating a preference for the aqueous media instead of the organic. A good solubility in water is needed in order to favor the transport phenomena for the compound to reach the active site; however, a very low lipophilicity might jeopardize the ability of such to traverse through the cell membrane [45,46]. Thus, the compounds with the highest absolute CLog*P* values (over |2|) have the least bioactivity (**3** and **5**), while the most active ones **4** (−1.153), **6** (−1.849) and **2** (−1.941) have lower CLog*P* values. In the case of compound **4**, the less affinity to water is joined to the presence of the benzoyl group which may have a positive effect on the bioactivity by release of this group by enzymatic transformation to benzoic acid inside the cell. As mentioned above, it has been reported that standalone benzoic acid has phytotoxic properties. The much lower activity of compound **1** when compared with **4** might be caused by two factors: their lipophilicity (CLog*P*: +1.885) and a possible synergistic effect with **4** after metabolization.

**Figure 5:**
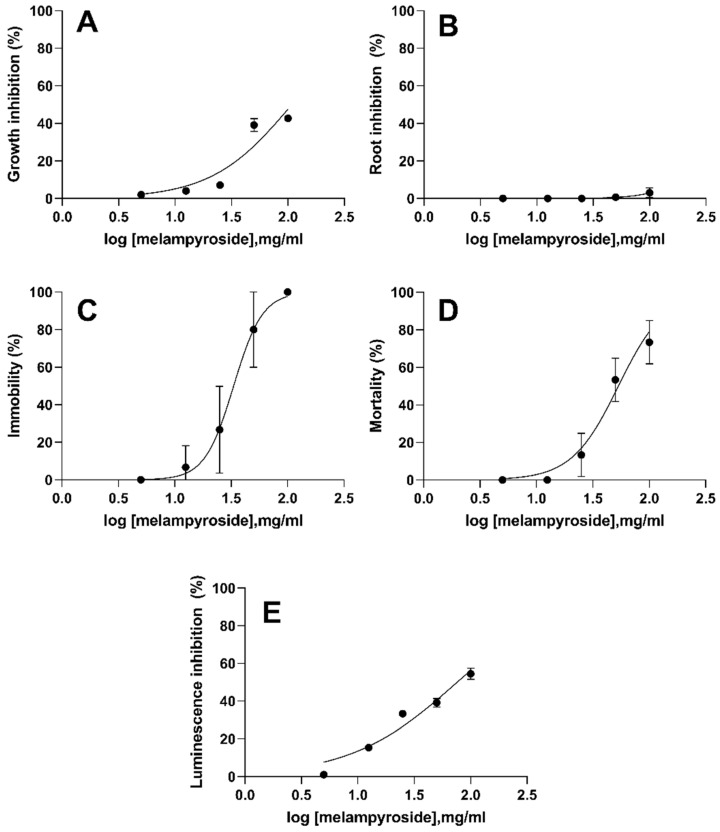
Concentration–response curves of melampyroside (**4**) for *R. subcapitata* (**A**), *L. sativum* (**B**), *D. magna* (**C**), *C. elegans* (**D**) and *A. fischeri* (**E**). Error bars correspond to 95% confidence intervals. Dotted lines represent the fitting to the effect equation.

The ecotoxicological tests are considered a valuable tool for preliminary toxicity screening of compounds, especially plant extracts [47]. The ecotoxicity of compound **4** was determined in three aquatic and two terrestrial organisms with different concentrations starting from 100 µg/mL, namely the effective concentration used for *O. cumana*. The influence of compound **4** on the observed effect in *R. subcapitata, L. sativum, D. magna, C. elegans* and *A. fischeri,* was shown in Figure 5.

The results of ecotoxicity showed significant differences in the sensitivity of tested organisms. The differences were reflected in the sensitivity of the plant species and the other organisms to compound **4**. While melampyroside showed the highest toxicity to daphnia (24 h EC_50_ = 33.26 µg/mL), nematodes (24 h EC_50_ = 57.23 µg/mL) and bacteria (EC_50_ 30′ = 76.05 µg/mL), it was less toxic for plant species, such as algae and macrophytes with 72 h EC_50_ ≥ 100 µg/mL. It is noteworthy that, when compared the effect in *L. sativum* by concentration, the difference was not statistically significant from 5 µg/mL to 100 µg/mL, and the growth observed with these treatments was not significantly different from the growth of control group (Figure 5B). One explanation for this different species-specific sensitivity to melampyroside may be related to the non-absorption of this compound by some plants suggesting a selectivity of melampyroside to inhibit radicle growth of *O. cumana* [48].

Considering the EU-Directive 93/67/ECC (EC, 1996) [49] (whereby EC_50_ values < 1.0 µg/mL were considered highly toxic; 1.0–10 µg/mL are considered toxic, 10–100 µg/mL were classified as slightly toxic and above 100 µg/mL were non-toxic), the response of the investigated organisms revealed that compound **4** had little or no toxicity. In contrast to other compounds that are exceedingly toxic, melampyroside could be considered as potential antiparasitic weed agent with an optimal toxicity/selectivity ratio [50,51].

## 3. Conclusions

Ethyl acetate extracts from different organs of *B. trixago* exhibited significant levels of growth inhibition on radicles of *O. cumana* at extract concentration as low as 100 µg/mL. Subsequently, we isolated and identified the active compounds contained in the ethyl acetate extract of aerial vegetative organs. We found three iridoid glycosides bartsioside (**2**), melampyroside (**4**) and mussaenoside (**6**) with growth inhibition activity in the radicles of *O. cumana*. The radicle of *Orobanche* species is a parasitic organ that grows towards the host and upon host contact, it allows infection. The use of allelochemicals to inhibit the normal growth of *Orobanche* radicles inhibits crop infection and, as a consequence, the death of this parasitic weed. Ecotoxicological tests carried out on compound **4** (the most abundant and phytotoxic iridoid isolated from ethyl acetate extract) showed little or no toxicity according to the EU Directive 93/67/ECC [49]. Plant species-specific phytotoxicity is a desirable trait in the development of novel molecules with herbicidal action to satisfy the principle of pesticide selectivity recommended for integrated pest management [52]. Future studies should determine, at the molecular level, the mode of action of the active iridoid glycosides on the radicles of *O. cumana.*

## 4. Materials and Methods

### 4.1. General Experimental Procedures

A JASCO P-1010 digital polarimeter (Tokyo, Japan) was used to measure the optical rotations. ^1^H NMR spectra were recorded at 400/100 MHz on a Bruker 400 Anova Advance (Karlsruhe, Germany) spectrometer or at 500/125 MHz on a Varian Inova 500 (Palo Alto, CA, USA). The spectra were recorded using CDCl_3_ or CD_3_OD and the same solvents were used as internal standards. Column chromatography (CC) was performed using silica gel (Kieselgel 60, 0.063–0.200 mm, Merck, Darmstadt, Germany). Thin-layer chromatography (TLC) was performed on analytical and preparative silica gel plates (Kieselgel 60, F_254_, 0.25 and 0.5 mm, respectively, Merck, Darmstadt, Germany). The spots were visualized via exposure to UV light (254 nm) and/or iodine vapours and/or by spraying first with 10% H_2_SO_4_ in MeOH and then with 5% phosphomolybdic acid in EtOH, followed by heating at 110 °C for 10 min. Electrospray ionization mass spectra (ESIMS) were performed using the LC/MS TOF system AGILENT 6230B (Agilent Technologies, Milan, Italy), HPLC 1260 Infinity. Sigma-Aldrich Co. (St. Louis, MO, USA) supplied all the reagents and the solvents.

### 4.2. Plant Material

Plants of two populations of *Bellardia trixago*—a white-flowered population and yellow-flowered population—were harvested at the phenological stage of flowering in spring of 2021 in Cordoba, southern Spain (coordinates 37.856 N, 4.806 W, datum WGS84). *Bellardia trixago* plants were immediately carried to the laboratory, and the plants were separated into three compartments: flowers, aerial green organs (stems and leaves) and roots. Each compartment was immediately frozen with liquid nitrogen, stored at −80 °C, subsequently lyophilized and the dry material stored in the dark at 4 °C until use. *Orobanche* seeds were collected from mature plants of *O. cumana* infecting sunflowers in southern Spain. Dry parasitic seeds were separated from capsules using winnowing combined with a sieve of 0.6 mm mesh size and then stored dry in the dark at room temperature until use for this work.

### 4.3. Extractions of *Bellardia trixago* Organs

A total of 3.0 g of each *B. trixago* organs were extracted, following a previously reported protocol often used for the extraction of plant material [53,54], in order to perform a preliminary activity screening against parasitic plants. In particular, the flowers, green organs and roots of each population were extracted separately by H_2_O/MeOH (1/1, *v*/*v*), under stirred conditions at room temperature for 24 h. The hydroalcoholic suspensions were centrifuged at 7000 rpm and extracted with *n*-hexane (3 × 50 mL), CH_2_Cl_2_ (3 × 50 mL), and after removing methanol under reduced pressure, with EtOAc (3 × 50 mL). The yield of each extract is reported in SI (Table S1).

### 4.4. Isolation and Identification of Metabolites from *Bellardia trixago* Green Organs of the White-Flowered Population

White green organs (189.0 g) were extracted (1 × 500 mL) by H_2_O/MeOH (1/1, *v/v*), under stirred conditions at room temperature for 24 h, the suspension centrifuged, and the supernatant extracted by *n*-hexane (3 × 300 mL) and successively with CH_2_Cl_2_ (3 × 300 mL) and, after removing methanol under reduced pressure, with EtOAc (3 × 200 mL). The residue (1.45 g) of EtOAc organic extract was purified by CC eluted with CH_2_Cl_2_/MeOH (8.5/1.5, *v/v*) yielding nine homogeneous fractions (F1-9), as reported in Scheme S1. The residue (54.6 mg) of F3 was purified by TLC eluted with EtOAc/MeOH/H_2_O (9/0.75/0.25, *v/v/v*), yielding six groups of homogeneous fractions (F3.1-F3.6). F3.1 was identified as benzoic acid (**1**, 10.8 mg) and F3.5 as melampyroside (**4**, 8.4 mg). The residue (584.7 mg) of fraction F4 yielded pure melampyroside (**4**). The residue (77.9 mg) of F5 was purified by TLC eluted by CH_2_Cl_2_/EtOAc/MeOH (2/2/1, *v/v/v*), yielding two homogeneous fractions. The first fraction of the latter purification yielded a further amount of melampyroside (**4**, 20.4 mg, for a total of 613.5 mg). The residue (498.4 mg) of F6 was purified by CC eluted with CH_2_Cl_2_/EtOAc/MeOH (2/2/1, *v/v/v*), yielding seven fractions (F6.1-F6.7). The residue (36.5 mg) of F6.4 was further purified by reverse-phase TLC eluted with MeCN/H_2_O (4/6, v/v), yielding gardoside methyl ester (**5**, 2.0 mg), bartsioside (**2**, 13.9 mg), and mussaenoside (**6**, 6.1 mg). The residue (64.7 mg) of F7 was purified by TLC eluted with EtOAc/MeOH/H_2_O (8.5/1/0.5, *v/v/v*) giving further amount of mussaenoside (**6**, 2.3 mg, for a total of 8.4 mg) and aucubin (**3**, 12.4 mg).

Benzoic acid (**1**): ^1^H NMR spectrum (Figure S1) was in agreement with data previously reported [55]. ESI MS (-) *m/z*: 121 [M − H]^−^.
Bartsioside (**2**): [α]_D_^22^-71.9 (c 0.64, MeOH) [lit. [56]: [α]_D_^25^-86.4 (c 0.5 MeOH)]. ^1^H NMR spectrum (Figure S2) was in agreement with data previously reported [57]. ESI-MS (+), *m/z*: 330 [M + H]^+^.
Aucubin (**3**): [α]_D_^22^-89.8 (c 1.0, MeOH) [lit. [27]: [α]_D_^26^-92.8 (c 0.27, MeOH)]. ^1^H NMR spectrum (Figure S3) was in agreement with data previously reported [17,58,59]. ESI-MS (+), *m/z*: 347 [M + H]^+^.
Melampyroside (**4**): [α]_D_^22^-69.6 (c 0.79, MeOH) [lit. [27]: [α]_D_^26^-52.9 (c 0.31, MeOH)]. ^1^H and ^13^C NMR spectra (Figures S4 and S5) were in agreement with data previously reported [11,49], while its NOESY spectrum is reported in Figure S6. ESI MS (+) *m/z*: 451 [M + H]^+^.
Gardoside methyl ester (**5**): [α]_D_^22^-49.8 (c 0.20, MeOH) [lit. [28]: [α]_D_^20^-46 (c 0.3, MeOH)]. ^1^H NMR spectrum (Figure S7) was in agreement with data previously reported [11,22]. ESI-MS (+), *m/z*: 389 [M + H]^+^.
Mussaenoside (**6**): [α]_D_^22^-81.3 (c 0.30, MeOH) [lit. [27]: [α]_D_^26^-77.9 (c 0.32, MeOH)]. ^1^H NMR spectrum (Figure S8) was in agreement with data previously reported [17,59,60,61]. ESI-MS (+), *m/z*: 391 [M + H]^+^.

### 4.5. Bioactivity on Parasitic Weed Seeds

Allelopathic effects of each *B. trixago* extracts and isolated compounds were tested on *Orobanche* radicle growth according to previous protocols [62]. Seeds of *Orobanche cumana*, were surface-sterilized by immersion in 0.5% (*w/v*) NaOCl and 0.02% (*v/v*) Tween 20, for 5 min, rinsed thoroughly with sterile distilled water, and dried in a laminar airflow cabinet. Approximately 100 seeds of *Orobanche* seeds were placed separately on 9 mm-diameter glass fiber filter paper disks (GFFP) (Whatman International Ltd., Maidstone, UK), moistened with 50 μL of sterile distilled water, and placed in incubators at 23 °C for 10 days inside Parafilm-sealed Petri dishes, to allow seed conditioning. Then, GFFP disks containing conditioned *Orobanche* seeds were transferred onto a sterile sheet of filter paper and transferred to new 9 cm sterile Petri dishes. Stock solutions of each *B. trixago* extract and isolated metabolite were respectively dissolved in dimethyl sulfoxide and subsequently individually diluted to 100 μg/mL using an aqueous solution of GR24 (10^−6^ M). The final concentration of dimethyl sulfoxide was 2% in all test treatments. For each assay, 50 μL aliquots of each sample were applied to GFFP discs containing conditioned *Orobanche* seeds. Triplicate aliquots of a treatment only containing GR24 and 2% dimethyl sulfoxide was used as a control. Treated seeds were incubated in the dark at 23 °C for 7 days and radicle growth was determined for each GFFP disc, using a stereoscopic microscope (Leica S9i, Leica Microsystems GmbH, Wetzlar, Germany). For the characteristic of radicle growth, the value used was the average of 10 randomly selected radicles per GFFP disc [63]. The percentage of radicle growth inhibition of each treatment was then calculated relative to the average radicle growth of control treatment.

### 4.6. Molecular Modelling

CLog*P* were calculated using ChemOffice v20.1 (PerkinElmer, Waltham, MA, USA) by means of the appropriate tool in ChemDraw Professional [64].

### 4.7. Ecotoxicity Analysis on Melampyroside

The ecotoxicological tests were carried out on green freshwater algae *Raphidocelis subcapitata*, macrophyte *Lepidium sativum*, water flea *Daphnia magna*, nematode *Caenorhabditis elegans* and bacterium *Aliivibrio fischeri* to expand the range of endpoints due to differences in species sensitivity and exposure. Testing on *R. subcapitata* was performed using as endpoint the algal growth inhibition after 72 h of exposure and was based on ISO 8692:2012 [65]. The algal density was determined by spectrophotometric analysis (DR5000, Hach Lange GbH, Weinheim, Germany). Ecotoxicity tests were carried out in triplicate, at 25 ± 1 °C with constant illumination of 6700 lux. Testing on *L. sativum* was performed according to ISO 11269-1:2012 [66] considering germination and root elongation as endpoint after 72 h. Seeds (n = 10) were exposed in triplicate in Petri dishes and incubated at 25 ± 1 °C in darkness. Daphnia magna test was conducted according to UNI EN ISO 6341:2013 [67] and the endpoint evaluated was the immobilization after 24 h. Daphnids (less than 24 h old) were exposed to the samples at 20 ± 2 °C, in darkness without feeding. Testing on *C. elegans* was carried out, with a few modifications, according to the ASTM E2172-01 Standard Method (2014) using the 24 h mortality endpoint. The test was performed using age-synchronous adult nematodes exposed at 20 °C to compound **4**, without feeding. Testing on *A. fischeri* was based on ISO 11348-3:2007 [68] and the inhibition of the bioluminescence of the bacterium after 30 min of exposure was measured as endpoint. The test was performed using Microtox^®^ Model 500 (M500) analyzer with osmotic adjustment solution (OAS) at 15 ± 1 °C.

### 4.8. Data Analyses

All bioassays were performed using a completely randomized design. Percentage data in *Orobanche* assays were approximated to normal frequency distribution by means of angular transformation and subjected to analysis of variance (ANOVA) using SPSS software for Windows (SPSS Inc., Chicago, IL, USA). The significance of mean differences among treatments was evaluated by the Tukey test. The null hypothesis was rejected at the level of 0.05. Results of ecotoxicological tests were given as the mean of effect ± standard error. Median effect concentrations EC_50_, EC_20_ and EC_5_ were calculated as mean values and relative 95% confidence limit values for compound **4**. Statistical analysis was carried out via XLSTAT and GraphPad Prism 2.5. (Systat Software, San Jose, CA, USA).

## Figures and Tables

**Table 1:** Calculated Log*P* for compounds (**1**–**6**).

	1	2	3	4	5	6
CLog*P*	1.885	−1.941	−4.028	−1.153	−2.133	−1.849

## Data Availability

The data presented in this study are available on request from the corresponding author.

## Supplementary Materials

The following are available online at https://www.mdpi.com/article/10.3390/toxins14080559/s1, Table S1: Extract weights of organic extracts obtained from different *Bartsia trixago* organs; Scheme S1: Purification scheme of EtOAc extract of *B. trixago* white green organs; Figure S1: ^1^H NMR spectrum of benzoic acid, **1** (CDCl_3_, 400 MHz); Figure S2: ^1^H NMR spectrum of bartsioside, **2** (MeOD, 500 MHz); Figure S3: ^1^H NMR spectrum of aucubin, **3** (MeOD, 400 MHz); Figure S4: ^1^H NMR spectrum of melampyroside, **4** (MeOD, 400 MHz); Figure S5: ^13^C NMR spectrum of melampyroside, **4** (MeOD, 100 MHz); Figure S6: NOESY spectrum of melampyroside, **4** (MeOD, 400 MHz); Figure S7: ^1^H NMR spectrum of gardoside methyl ester, **5** (MeOD, 400 MHz). Figure S8: ^1^H NMR spectrum of mussaenoside, **6** (MeOD, 500 MHz).
