# Supplementary material for: Iridoid Glycosides Isolated from Bellardia trixago Identified as Inhibitors of Orobanche cumana Radicle Growth
Source: Toxins (Basel). 2022 Aug 17;14(8):559. doi: 10.3390/toxins14080559 (PMC9414233; doi:10.3390/toxins14080559)
Supplement: Supplementary file 1 [file toxins-14-00559-s001.zip › toxins-1843539-supplementary.pdf]

Supplementary Materials: Iridoid Glycosides Isolated from *Bellardia trixago* Identified as Inhibitors of *Orobanche cumana* radicle growth

Gabriele Soriano, Antonietta Siciliano, Mónica Fernández-Aparicio, Antonio Cala Peralta, Marco Masi, Antonio Moreno-Robles, Marco Guida, and Alessio Cimmino

Supporting Information List

	Page
**Table S1.** Extract weights of organic extracts obtained from different *Bartsia trixago* organs.	2
**Scheme S1.** Purification scheme of EtOAc extract of *B. trixago* white green organs.	2
**Figure S1**. ^{1}H NMR spectrum of benzoic acid, **1** ($CDCl_3$, 400 MHz).	3
**Figure S2**. ^{1}H NMR spectrum of bartsioside, **2** (MeOD, 500 MHz).	3
**Figure S3**. ^{1}H NMR spectrum of aucubin, **3** (MeOD, 400 MHz).	4
**Figure S4**. ^{1}H NMR spectrum of melampyroside, **4** (MeOD, 400 MHz).	4
**Figure S5**. ^{13}C NMR spectrum of melampyroside, **4** (MeOD, 100 MHz).	5
**Figure S6**. NOESY spectrum of melampyroside, **4** (MeOD, 400 MHz).	5
**Figure S7**. ^{1}H NMR spectrum of gardoside methyl ester, **5** (MeOD, 400 MHz).	6
**Figure S8**. ^{1}H NMR spectrum of mussaenoside, **6** (MeOD, 500 MHz).	6

Table S1. Extract weights of organic extracts obtained from different *Bartsia trixago* organs.

***Bartsia trixago* organ**	***n*-Hexane extract weight**	**CH_2Cl_2 extract weight**	**EtOAc extract weight**
White flowers	2.79 mg	12.11 mg	27.28 mg
White roots	1.24 mg	3.39 mg	13.39 mg
White green organs	1.00 mg	10.52 mg	44.67 mg
Yellow flowers	1.22 mg	10.13 mg	21.00 mg
Yellow roots	1.42 mg	3.78 mg	13.85 mg
Yellow green organs	2.74 mg	19.35 mg	11.54 mg

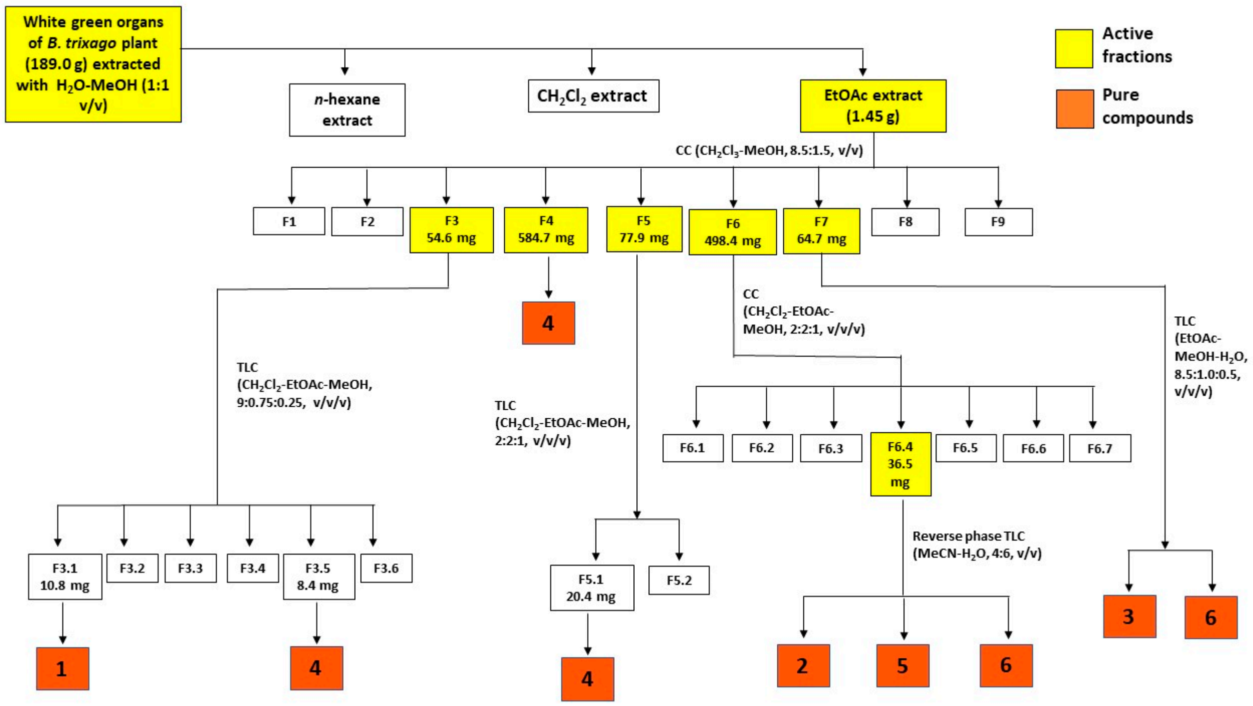

Scheme S1. Purification scheme of EtOAc extract of *B. trixago* white green organs.

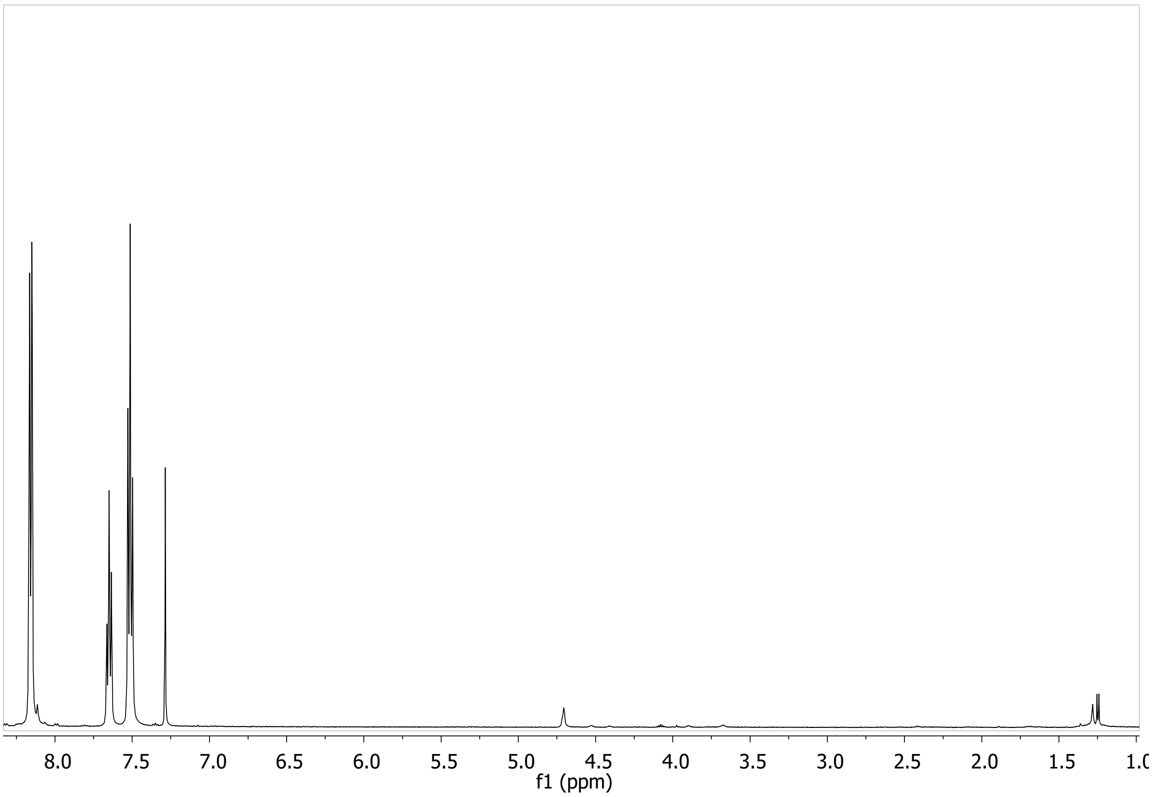

Figure S1. ^{1}H NMR spectrum of benzoic acid, **1** ($CDCl_3$, 400 MHz).

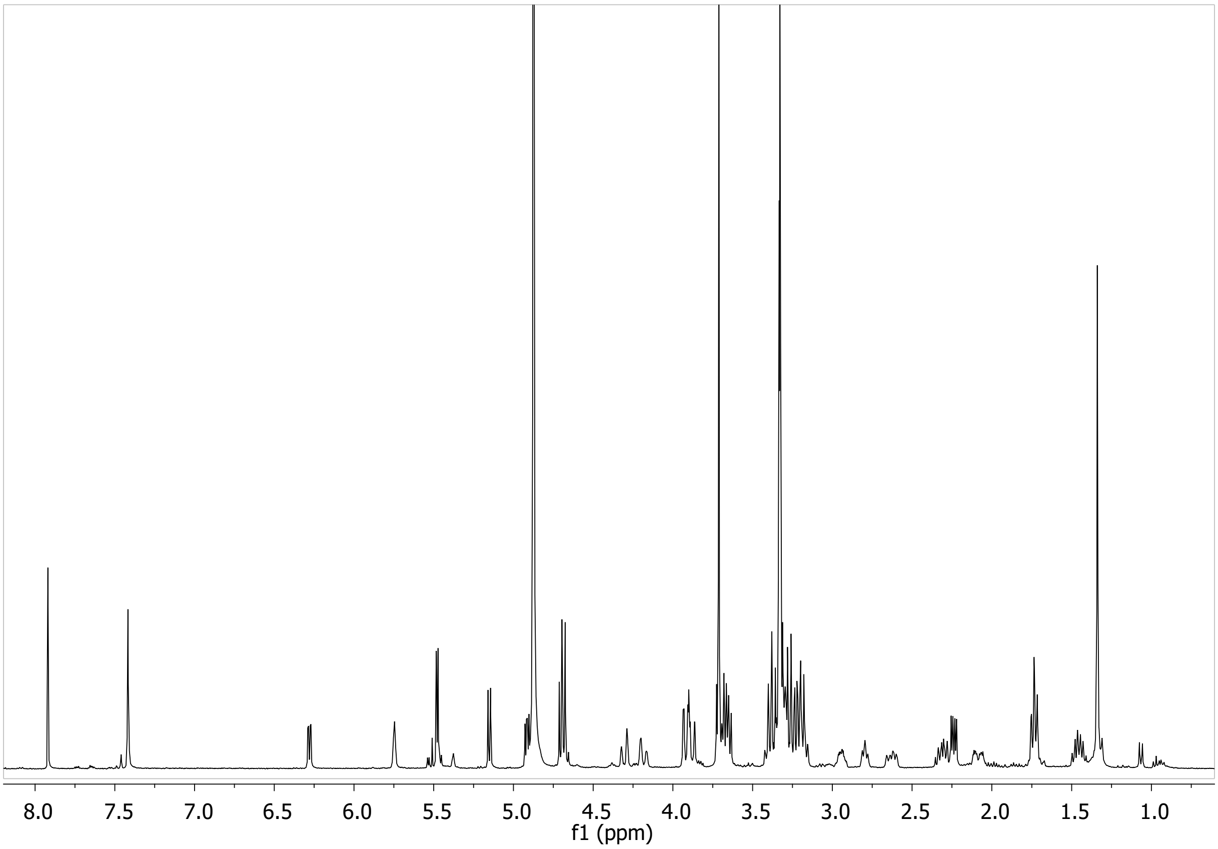

Figure S2. ^{1}H NMR spectrum of bartsioside, **2** (MeOD, 500 MHz).

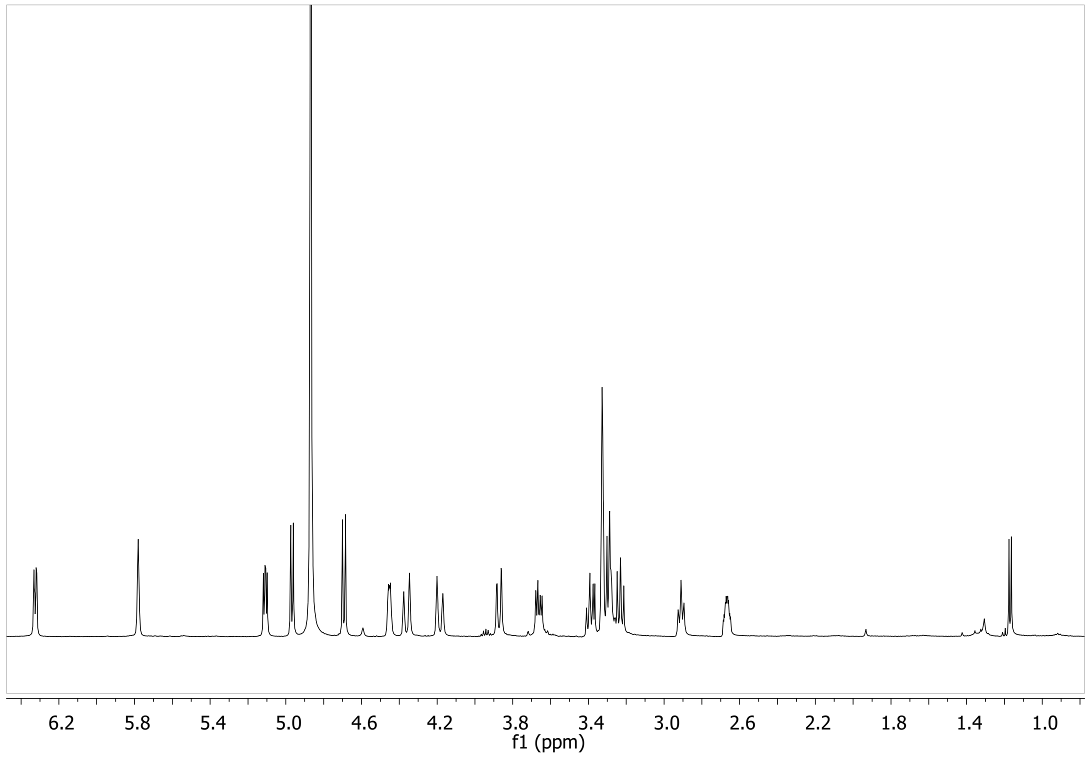

Figure S3. ^{1}H NMR spectrum of aucubin, **3** (MeOD, 400 MHz).

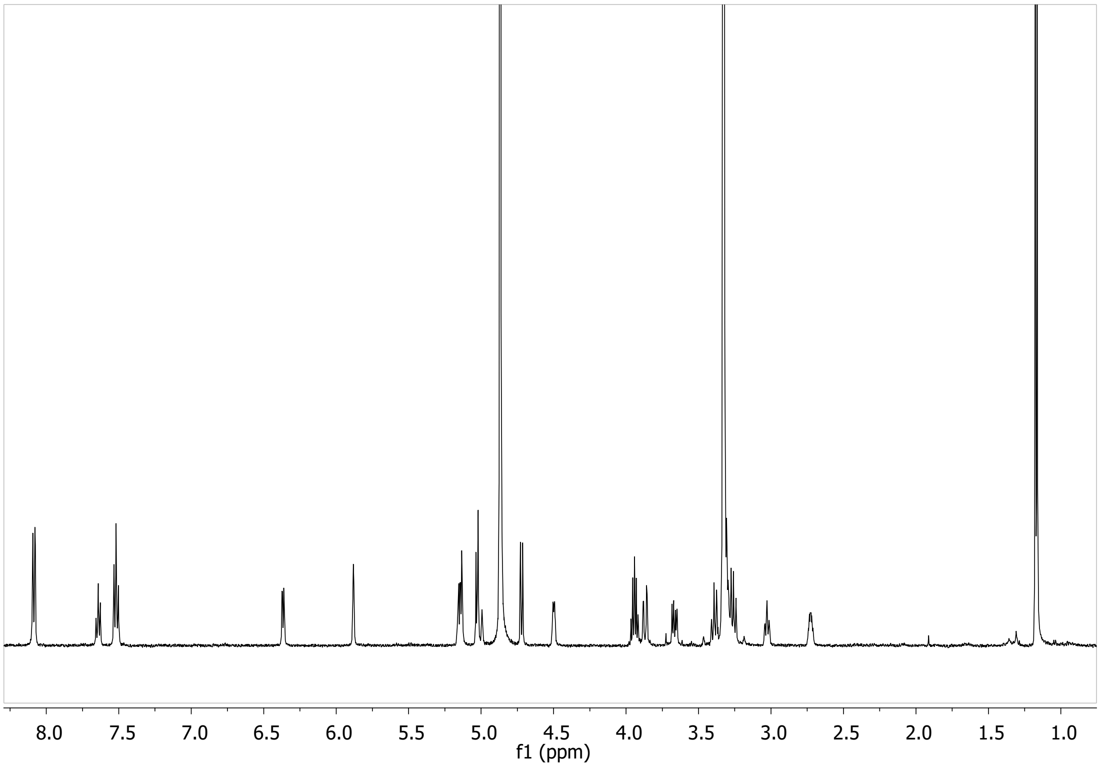

Figure S4. ^{1}H NMR spectrum of melampyroside, **4** (MeOD, 400 MHz).

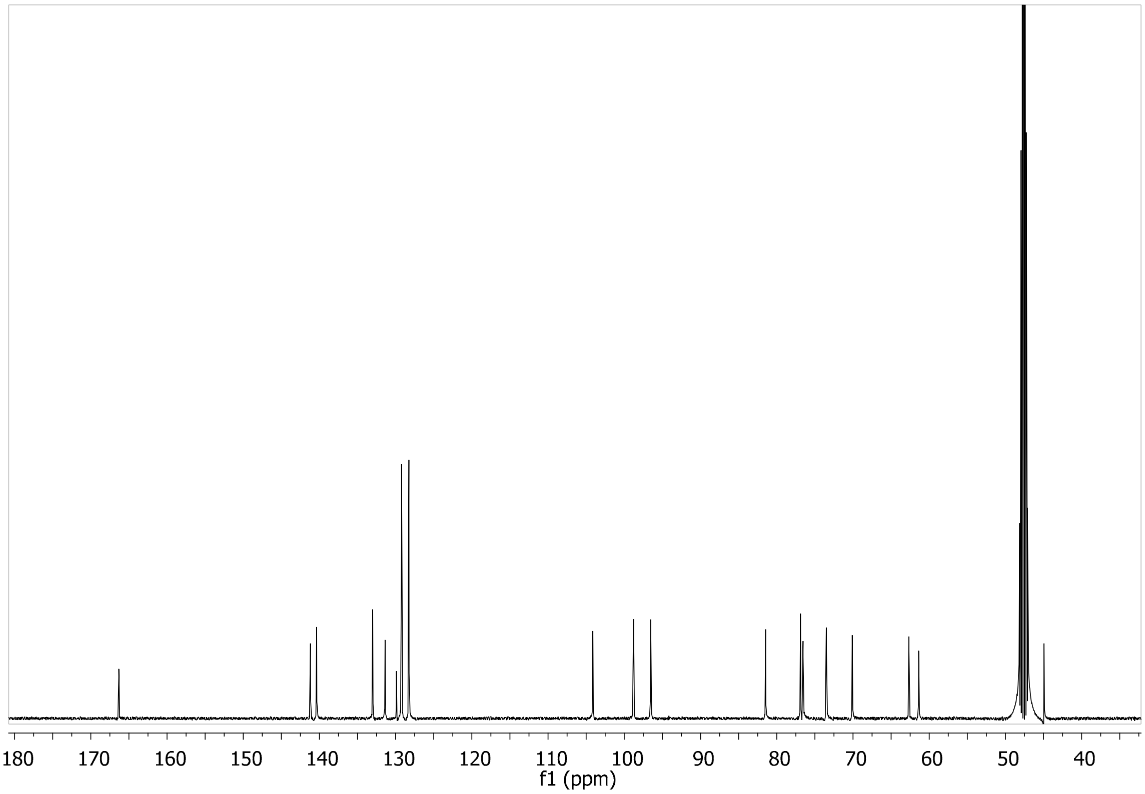

Figure S5. ^{13}C NMR spectrum of melampyroside, **4** (MeOD, 100 MHz).

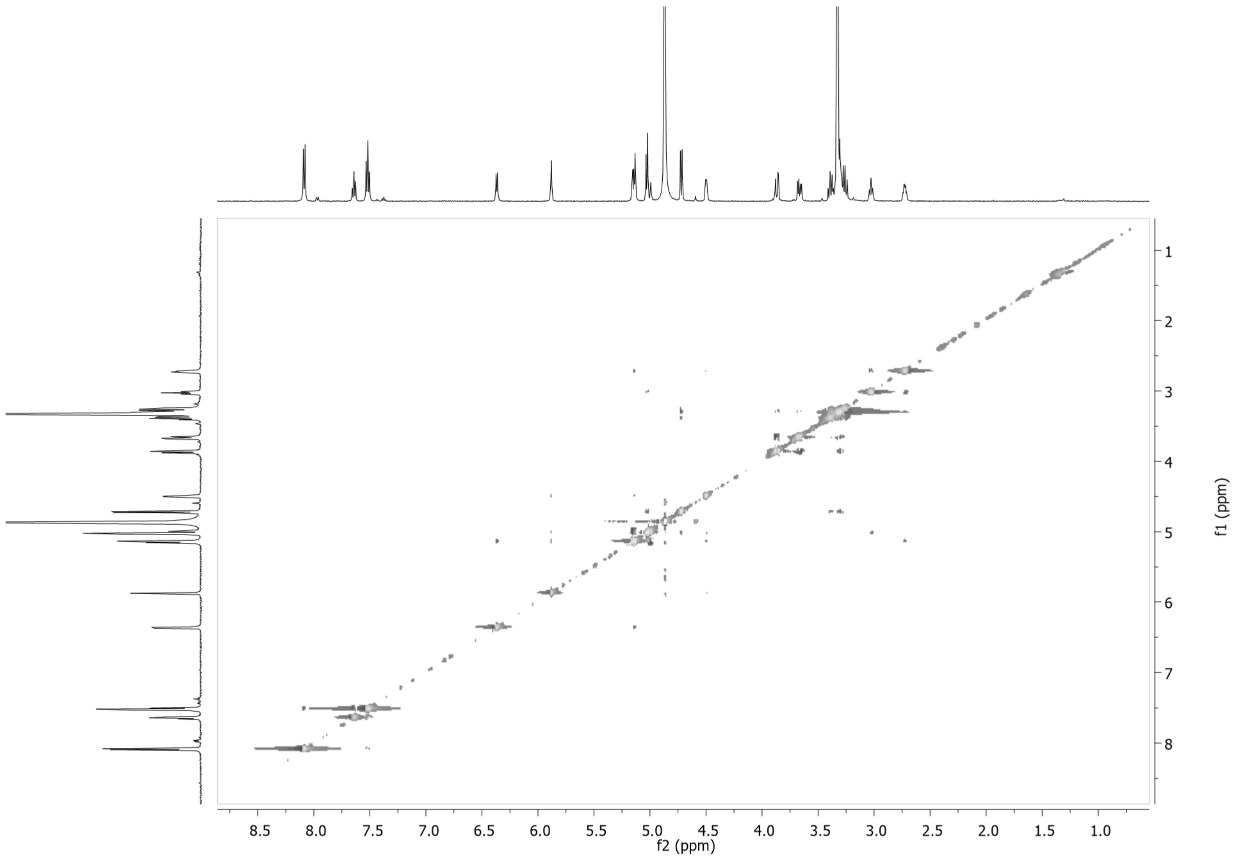

Figure S6. NOESY spectrum of melampyroside, **4** (MeOD, 400 MHz).

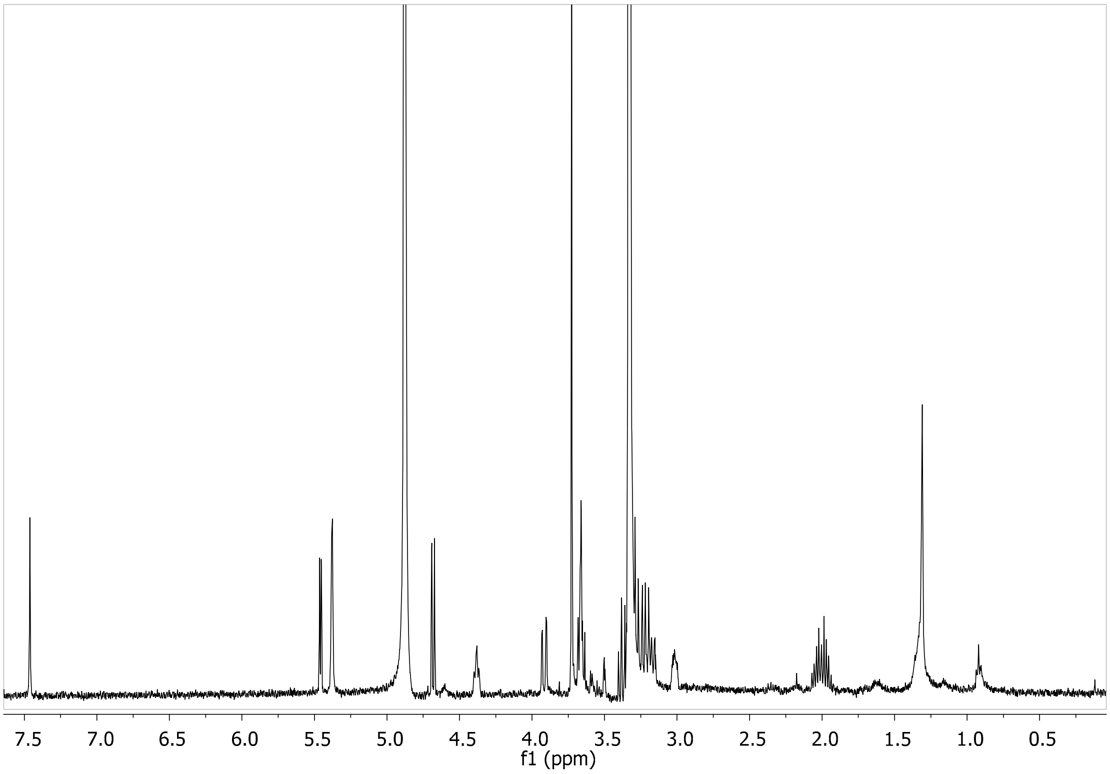

Figure S7. ^{1}H NMR spectrum of gardoside methyl ester, **5** (MeOD, 400 MHz).

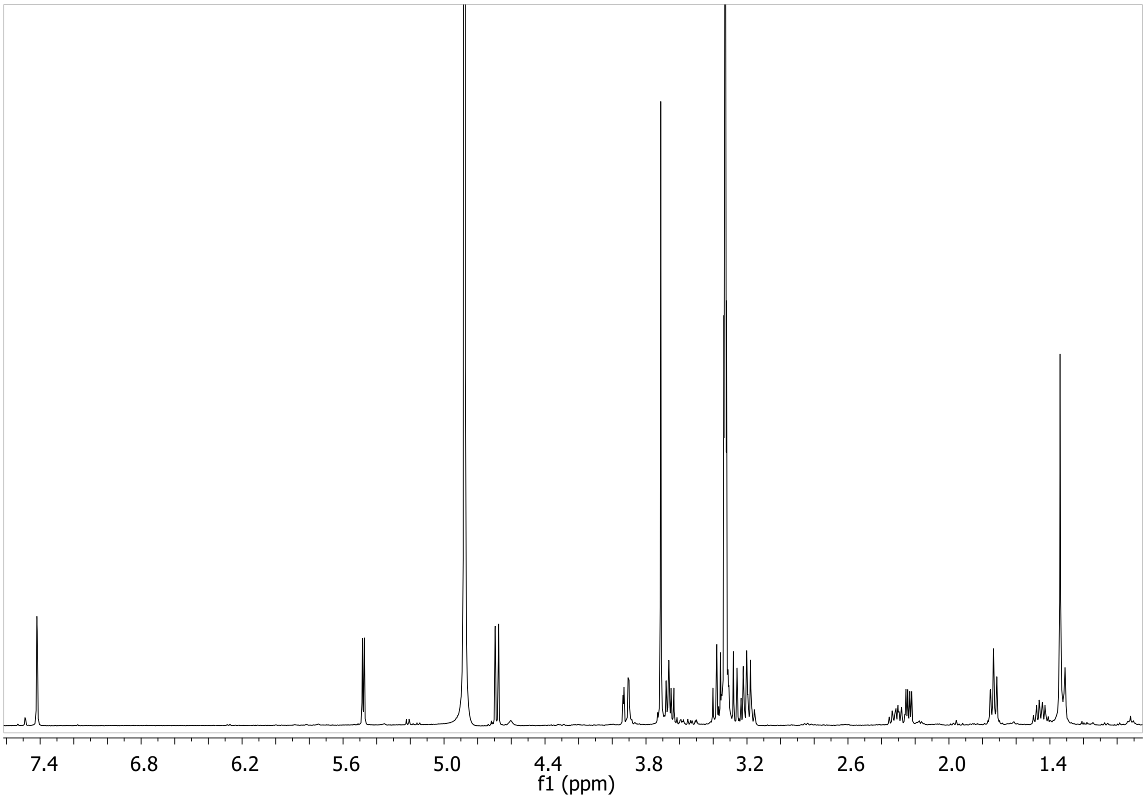

Figure S8. ^{1}H NMR spectrum of mussaenoside, **6** (MeOD, 500 MHz).
